# Post-Cracking Capacity of Glass Beams Reinforced with Steel Fibers

**DOI:** 10.3390/ma12020231

**Published:** 2019-01-11

**Authors:** Marco Corradi, Emanuela Speranzini

**Affiliations:** Department of Engineering, University of Perugia, Via G. Duranti, 93 06125 Perugia, Italy; emanuela.speranzini@unipg.it

**Keywords:** structural glass, steel fiber, bending behavior, numerical modelling

## Abstract

A study concerning the flexural behavior of glass beams reinforced with steel fibers is presented in this paper. Two types of steel fibers were used for reinforcement, made of high strength and stainless steel. The coupling effect of the two materials was studied in terms of energy dissipation and failure loads, by comparing the elastic limits and the post-elastic behaviors of the reinforced glass beams. Results demonstrated that it is possible to increase the overall structural safety of a steel fiber reinforced glass beam. The relationship between the bending force and deflections was initially linear, however, following the opening of first cracks in the glass, the reinforcement steel material was able to withstand the tensile stresses, governing the overall post-elastic phase.

## 1. Introduction

Glass is appreciated in civil engineering and architecture for its transparency. It has recently found widespread use in large structures. However, it is known that cracking can occur in glass for low tensile or bending stresses (spontaneous failure). This is often induced by the presence of flaws randomly distributed on the glass’s surface. The bending response of a glass beam is linear elastic until the opening of the first tensile cracks. Once triggered, these cracks propagate without any possibility of being stopped, given the low glass toughness, and leading to the collapse with the formation of a large number of fragments. For this reason, safety can be ensured by using glass sheets to form laminated glass, capable of retaining the fragments.

A large amount of research has been conducted on laminated glass [1,2], interlayer viscoelastic response [3,4], numerical analysis of glass structures [5], and experimental analysis of buckling phenomena due to bending and torsional loading [6,7]. Nevertheless, glass’s fragility and spontaneous failure require considering the post-cracking phase in its calculations and analyses [8,9]. This implies the need for a new design philosophy, known as “Fail safe”, which foresees the failure of individual structural elements [10]. It is therefore possible for a laminated glass structure to retain an adequate resistant capacity, preventing a fragile failure.

In fragile materials, the strength alone [11] does not deliver a resilient system. To increase robustness and ductility, glass structures need to be reinforced with different materials. These can provide the needed tensile strength that glass does not typically exhibit. Therefore, the addition of a reinforcing material improves the flexural behavior; the bending capacity is increased, alternative stress paths are possible and structural safety is also enhanced.

For this purpose, many types of reinforcement can be used. The application of a composite reinforcement can prevent a brittle failure and increase the beam load-capacity. An overview of recent developments in reinforcement of glass structures is presented in Martens et al. [12]. Composite glass structures were classified in terms of reinforcing materials: metals, Glass Fiber Reinforced Polymers (GFRPs), timber, Reinforced Concrete (RC), and plastics. In more recent publications, glass structures were reinforced with steel bands [13,14,15], steel profiles [16,17,18], GFRP sheets [19,20,21], timber [22,23], ultra-high-performance RC [24], and ductile polycarbonate foils [25,26]. Other prototypes of glass beams were reinforced with Carbon Fiber Reinforced Polymers (CFRP) sheets or rods [27,28]. Reinforcements were typically applied to the beam’s bottom as a tensile resistant material. Pre-stressed steel elements were also used as reinforcement for glass beams [29].

Among the types of reinforcements tested in the past, the most interesting results were obtained with steel profiles and steel fibers [13,14,15,16,17,18]. These beams exhibited a prolonged post-cracking phase and a greater ductility compared to the beams reinforced with pultruded GFRP profiles. Reinforcements with greater stiffness (=E·I_n_, where E and I_n_ are the Young’s modulus and the second moment about the neutral axis, respectively) are generally more effective. For example, U-shaped steel profiles should prevail over horizontally placed profiles (lamina). This has a positive effect during the post-cracking phase, as a stiff reinforcement is able to absorb the tensile stresses without a significant reduction of the beam load-capacity. Finally, it is worth noting that the behavior of the reinforced beams is also influenced by the glass type (float, hardened, or tempered glass).

The bending behavior of hardened or tempered glass beams, in terms of displacements, is similar to float glass with the following differences: (1) the linear load-displacement relationship is prolonged, given the higher tensile strength of the hardened and tempered glass; (2) the post-cracking phase is smaller. This can be explained by considering that the post-cracking behavior depends on the interlocking between the glass fragments. This depends on the shape and size of the fragments. Various experiments carried out in the laboratory demonstrated that fragment interlocking does not occur in cracked tempered glass, (due to the formation of rounded-edge fragments), while it has an effect for cracked hardened glass and float glasses where the fragments are edgy [18,19].

In a steel or a RC structure, ductility can develop at various levels: (1) intrinsic ductility linked to structural material [30], (2) local ductility corresponding to a localized plastic deformation, and (3) structural (or global) ductility associated with the plasticization of a single element of the structure. It is not possible to recognize an intrinsic ductility for glass since glass is given the linear elastic stress-strain relationship up to failure both in compression and in traction.

The application of a reinforcement, even if made up of a material able to undergo non-reversible changes of shape (plastic behavior), cannot provide the required ductility, as it can hardly exceed its yield strength during the linear elastic loading phase. Despite these limitations, after glass cracking, the tensile resistant characteristics of the reinforcement can guarantee a pseudo-plastic response.

This paper aims at proposing a method for reinforcing glass beams with unidirectional steel fibers. The analysis investigates the post-elastic behavior of steel fiber reinforced glass beams by taking into account appropriate strain energy parameters. Test results can be used to analyze typical failure modes involving steel fibers or glass substrates. The tensile stress migration from the glass to the fiber, inside the reinforced glass beam, can guarantee further load-capacity increment after glass cracking. The results of the experiment carried out in the laboratory on steel fiber reinforced glass beams are presented. Reinforced beams were tested in flexure (four-point bending test) and their structural response was studied in terms of dissipated energy and load-capacity in the elastic and post-elastic phases.

## 2. Materials and Work Methods

### 2.1. Materials Tested

The behavior of a steel fiber reinforced glass beam is mainly based on an efficient connection between the two materials, established by the bonding between the reinforcement and the glass surfaces. Adhesion phenomena are of critical importance in order to achieve a high performance of the reinforced system. Speranzini et al. [31] found that delamination often occurs as a result of the detachment by peeling of the reinforcement.

#### 2.1.1. Resin and Glass

To connect the steel fibers to the bottom surface of the steel beams, a two-component transparent epoxy resin was used. The type of the resin was selected on the basis of the results obtained in previous experiments [31,32,33] carried out in the laboratory to study the adhesion between epoxy resin and glass. Table 1 shows the main physical and mechanical characteristics of the used epoxy resin.

Glass beams were made by overlapping 8 or 12 mm-thick glass sheets of annealed float glass according to the EN 572 standard [34] (Typical mechanical characteristics: weight density 2500 kg/m^3^, Young’s modulus 70 GPa, flexural strength 45 MPa, Poisson’s ratio 0.2). Tensile resistant material, used as reinforcement, is then bonded to the intrados of the glass beam by means of a two-component epoxy resin with high transparency, to cover the whole width.

#### 2.1.2. Steel Reinforcement

**Figure 1:**
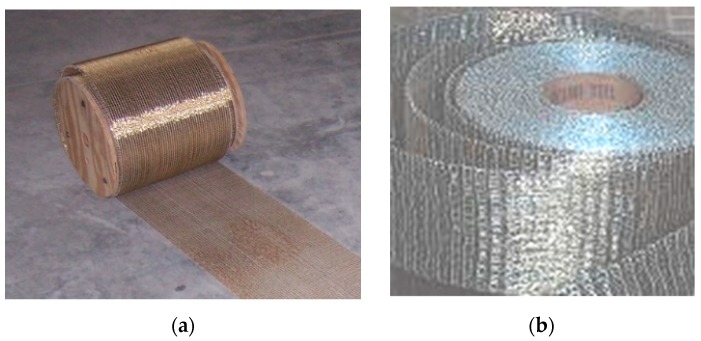
Steel fibers: (**a**) Ultra High Tensile Strength Steel (UHTTS) fibers; (**b**) stainless steel fibers.

Two types of unidirectional steel fibers were used to reinforce the glass beams: carbon Ultra High Tensile Strength Steel (UHTTS) and stainless steel fibers. The latter are corrosion resistant and can be used in aggressive environment. Both fibers were applied on the beam tension side using an epoxy resin. Figure 1 shows the two types of fibers and Table 2 reports the main mechanical properties of the fibers. Steel fibers were made of adjacent cords. Each cord was assembled by twisting small filaments together.

#### 2.1.3. Beam Geometry

**Figure 2:**
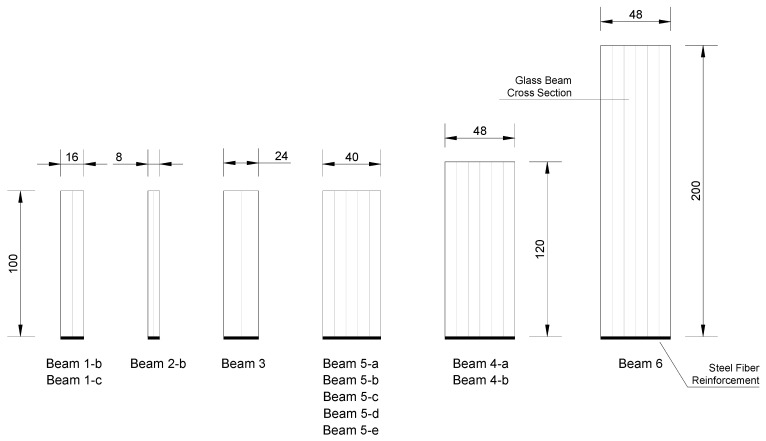
Dimensions of the cross sections of the steel fiber reinforced beams (dimensions in mm).

Six series of glass beams, for a total number of 35 specimens, were tested in this experiment. Beams differed in span length, cross section dimensions, type and amount (A_steel_) of steel fiber reinforcement. Figure 2 shows the dimensions of the cross sections and the location of the steel fiber reinforcement. The height of the glass beams was 100 mm (series Beam 1, 2 and 3), 120 mm (series Beam 4), and 200 mm (series Beam 6). These were tested over a span varying from 900 to 2800 mm. The geometric characteristics of the beams are summarized in Table 3.

### 2.2. Work Methods

#### 2.2.1. Test Method

**Figure 3:**
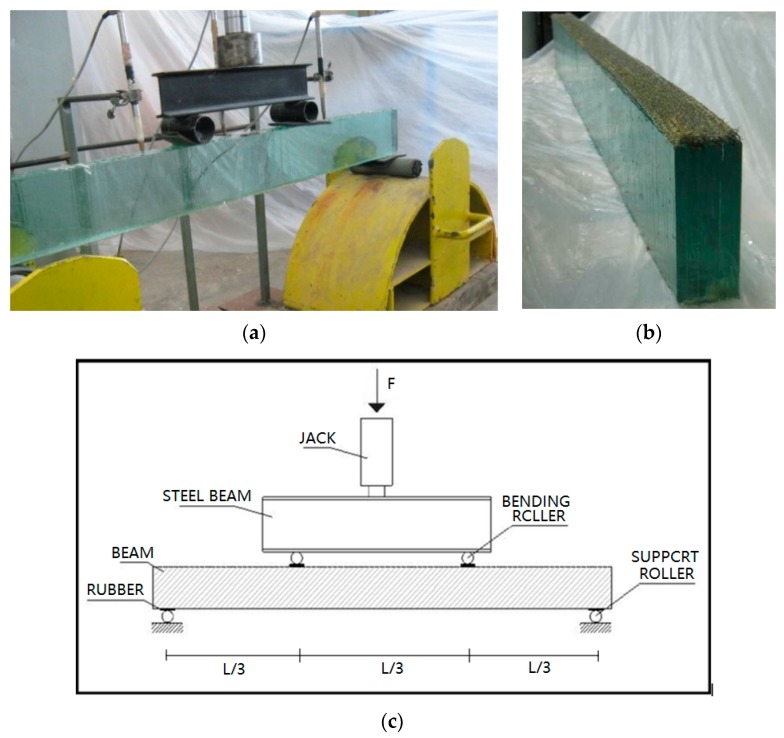
Glass-steel fibers and test setup: (**a**) test layout; (**b**) detail of a glass specimen; (**c**) Four-point bending test.

In this experiment, all specimens were tested in bending (Figure 3) in accordance with the UNI EN 1288-3 standard [35] (four-point bending). The test specimens were equipped with inductive transducers (Linear Variable Differential Transducers (LVDT)) in order to record the vertical displacement. The bending load was statically applied with a hydraulic jack anchored to the loading steel frame. All the acquisitions, including load and displacements, were automatically recorded by a data acquisition system. Special attention was given to the analysis of the post-cracking phase. The acquisition system recorded the magnitude of bending load, time and vertical displacements at 1/3, 1/2 (mid-span), and 2/3 of the beam span.

#### 2.2.2. Numerical Analysis

The post-cracking response was numerically studied, using a commercially available software (Ansys, Cannonsburg, PA, USA) [36]. A reinforced specimen from Series Beam 6 was modeled. Glass and epoxy resin were modelled using SOLID65 element in Ansys workbench. This is a 3-D element defined by eight nodes having three degrees of freedom at each node. SOLID65 is capable of cracking in tension and crushing in compression and allows the definition of a shear transfer coefficient that ranges from 0 to 1. This coefficient is defined for closed and open cracks: when it is equal to 0, it simulates a smooth crack (with a complete loss transfer capacity of shearing force), while it simulates a rough crack with no loss of transfer capacity when its value is 1. Steel fibers were modelled using SHELL41 that is a 3-D element defined by four nodes with three degrees of freedom at each node and having only membrane stiffness. Glass properties were: weight density 2.5 × 10^3^ kg/m^3^, Young’s modulus 70 GPa, Poisson ratio 0.21, tensile strength 45 MPa, and compressive strength 800 MPa. For the epoxy resin: weight density 1.08 × 10^3^ kg/m^3^, Young’s modulus 1760 MPa, Poisson ratio 0.29, tensile strength 30 MPa, compressive strength 40 MPa, and failure tensile strain 1.6%. The mechanical properties of Table 2 were used for the fibers.

Four-point bending tests were numerically conducted; to simulate the post-elastic behavior, the Young’s modulus was progressively reduced during the application of the load, after cracking. This was reduced to 7 GPa when the tensile stress reached the limit value of 45 MPa. Cracks initially opened between the two loading points (in the area where the bending moment is constant). Just before the beam failure, cracks also occurred near the end-supports.

## 3. Test Results

**Figure 4:**
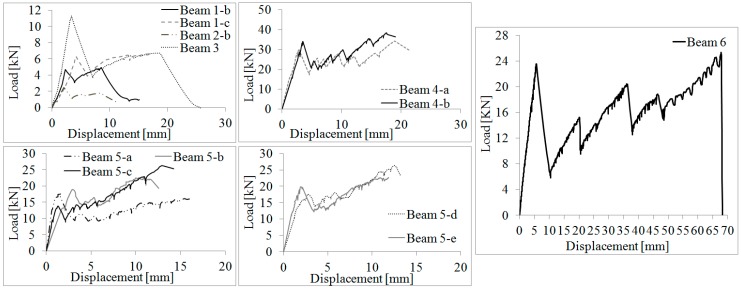
Load-displacement diagrams.

Test results are shown in Table 4. The bending load vs. mid-span displacement diagrams, representative of each type of beam, are plotted in Figure 4. It can be noted that the application of the steel reinforcement did not cause a notable increase of the bending capacity during the elastic phase—the limit loads for both unreinforced and reinforced beams are almost the same (Series 1 and 2). This can be explained by considering the similar Young’s moduli of the glass (70 GPa) and steel reinforcements (73.5 and 206 GPa for stainless and UHTSS fibers, respectively) and the small sectional area of the reinforcement. Furthermore, the low modulus of the epoxy adhesive may have partially compromised the reinforcement action during the elastic phase with slippage phenomena between glass and steel reinforcement. The structural response of unreinforced glass beams (Series Beam 1-a and Beam 2-a) was linear elastic up to failure and the limit elastic load coincided with the beam failure load.

**Figure 5:**
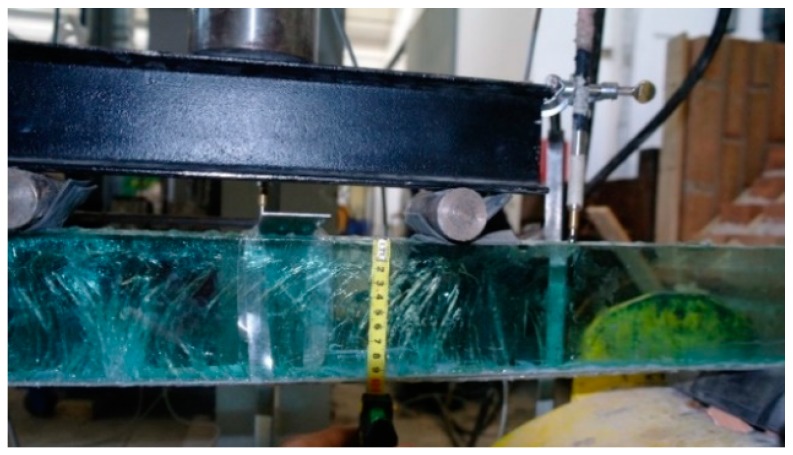
Typical crack pattern.

For reinforced beams, the behavior was significantly different and a post-elastic phase was recorded. The action of the reinforcement becomes more evident only when the reinforced glass beam starts cracking on the tension side. All reinforced specimens showed an elastic phase characterized by a linear stress-strain response until first cracks opened. This caused an abrupt reduction in the load-capacity. However, due to the reinforcement action, the capacity partially recovered and new cracks progressively opened until beam collapse occurred. The elastic phase was characterized by a linear stress–strain response. The values of the load and corresponding mid-span vertical displacement at the elastic limit are reported in Table 4 (columns No. 2 and 3). After the formation of the cracks, an abrupt reduction of the load-capacity occurred (the values of the residual load-capacity and corresponding mid-span displacement are given in Table 4 (columns No. 4 and 5). Thanks to the action of the steel reinforcement, the load capacity recovered (post-cracking phase) until failure (Table 4, columns No. 6 and 7 report failure loads and corresponding vertical deflections, respectively). The reinforcement steel fiber material guaranteed the development of this post-cracking phase; a sequence of load drops, as a consequence of a progressive glass cracking, and load recoveries followed through to collapse (Figure 5).

The load drops are described on a load vs. mid-span deflection graph by quasi-vertical segments while the load recoveries are represented by diagonal segments, the slope of which provided the cracked beam stiffness, which progressively decreased with the formation of new cracks. Table 4 also shows the stiffness values in the elastic (*K_el_*) and post-cracking phase (*K_post_*_-*el*_), given in terms of the slope of the line in the load vs. mid-span deflection relationship. For the post-elastic phase, this was calculated using two points given by the post-elastic and failure loads and corresponding deflections.

## 4. Discussion

The analysis of the results was performed in terms of failure loads and equivalent strain energy, using the experimental load vs. mid-span displacement diagrams. The area enclosed by the load- displacement curve can be associated to the total work, done by the applied bending load. It is worth noting that the energy values were calculated using mid-span deflections. The contact transducer (LVDT) was placed directly over the glass beam (the contrast was the ground floor of the lab). By doing this, the deformation of the spreader metal beam was not included in the value of the measured displacement.

For this purpose the following parameters were taken into consideration:

- For the cross sectional area of the tensile resistant material A_steel_ joined to glass, the ratio *S* was used:
  (1) $$S=n\cdot \frac{A_{steel}}{A_{glass}},$$
  where *n* is the ratio between the Young’s modulus of the two materials (steel fiber and glass)
  (2) $$n=\frac{E_{steel}}{E_{glass}},$$
- The ratio *R*, describing the increase of the failure load compared with the limit elastic load:
  (3) $$R=\frac{F_{fail}-F_{el}}{F_{el}},$$
- The ratio *T*, describing the vertical displacement at failure compared with the displacement at the elastic limit:
  (4) $$T=\frac{D_{fail}-D_{el}}{D_{el}},$$
- The ratio between the failure load and the load at the elastic limit:
  (5) $$V=\frac{F_{post-el}}{F_{el}},$$
- The ratio between *K_post-el_* (stiffness in the post-elastic phase) and *K_el_* (stiffness in the elastic field):
  (6) $$J=\frac{K_{post-el}}{K_{el}},$$
  *K_el_* is the slope of the line in elastic phase. This was calculated from the load vs. displacement diagram; *K_post-el_* is the stiffness in the post-elastic phase and was evaluated as the slope of the straight line between post-elastic load and failure load.
- Finally, the ratio between the failure load and the post-elastic load:
  (7) $$Q=\frac{F_{fail}}{F_{post-el}},$$

**Figure 6:**
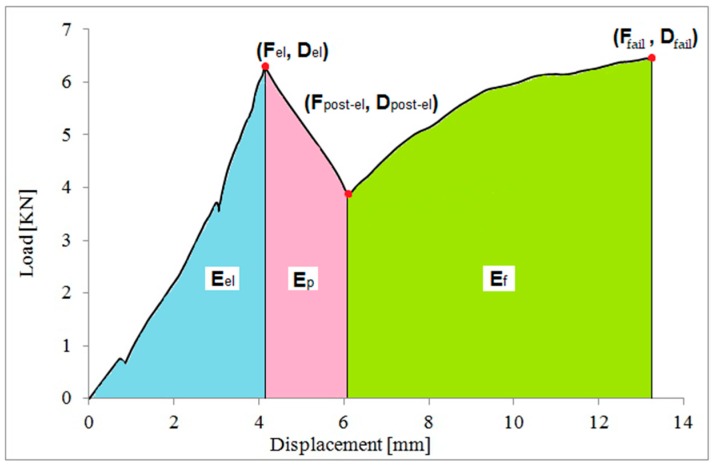
Determination of the energy contributions (load vs. mid-span deflections).

The values of the ratios computed for each tested series are shown in Table 5. The energy content of the different phases of the test can be assessed from the load vs. displacement diagram, as shown in Figure 6. The energy corresponding to the elastic, the post-elastic and the post-elastic phase are respectively *E_el_*, *E_p_* and *E_f_*. The addition of these three values represents the total energy *E_t_*.

The following dimensionless parameters Λ*_el_*, Λ*_p+f_* and Λ*_p/el_* were calculated:

(8) $$\Lambda_{el}=\frac{E_{el}}{E_{t}},$$

(9) $$\Lambda_{p+f}=\frac{E_{p}+E_{f}}{E_{t}},$$

(10) $$\Lambda_{p/el}=\frac{E_{p}}{E_{el}},$$

The values *E_el_*, *E_p_*, *E_f_* and *E_t_*, and the dimensionless parameter Λ*_el_*, computed for each beam series, are reported in Table 6.

### 4.1. Results in Terms of Failure Load

**Figure 7:**
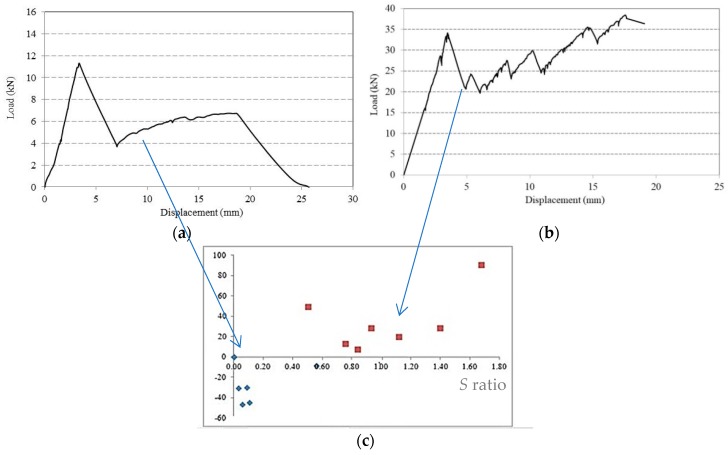
(**a**) Low *S* ratio: typical load-displacement diagram; (**b**) High *S* ratio: typical load-displacement diagram; (**c**) Diagram *R* ratio versus *S* ratio.

**Figure 8:**
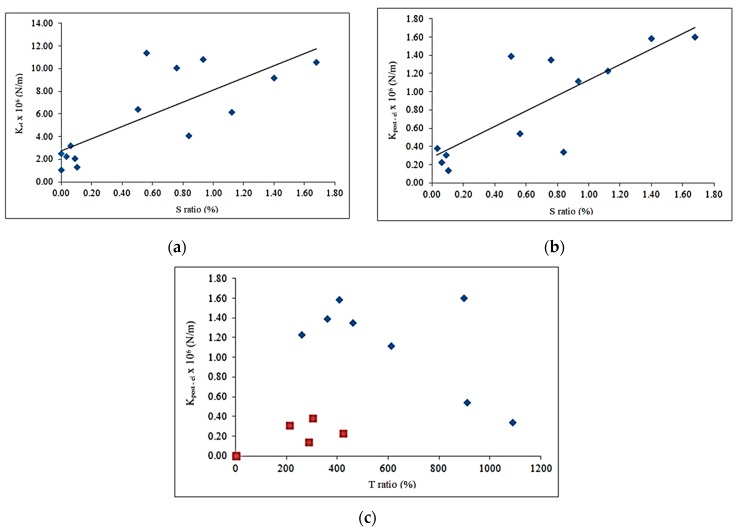
Diagrams of the variation of the stiffness.

The following observations regarding the failure, the elastic and the post-elastic loads can be drawn:

(a) It was initially observed that, for small values of the *S* ratio, the failure loads were typically smaller than the elastic limit loads; this occurred when the reinforcement wasn’t able to ensure an adequate post-elastic phase (Figure 7a,b). Figure 7c shows the experimental values of *R* versus *S* ratios: the blue dots represent the behavior of the beams with insufficient amount of steel reinforcement (negative values of *R* ratio), i.e., the failure load is smaller than elastic limit load. On the contrary, the red dots represent the behavior of the beams characterized by a failure load higher than the elastic limit load. The experimental results show that reinforced beams do not exhibit a satisfactory post-elastic behavior when the *S* ratio is less than 0.50%.

(b) The *V* ratio is an index of the load drop after the formation of the first crack and varies between 24.7% and 78.3% (Table 5).

(c) The *Q* ratio shows the reinforced cracked beam capacity to bear the load. The values of this ratio are typically in the percentage range 115–200%; this demonstrates that the reinforced beams are able to recover the load after cracking. The amount of the reinforcement area does not affect this ratio substantially.

(d) Stiffness values are given in Figure 8. Both the stiffness calculated in the elastic phase and in the post-elastic phase are linearly dependent with the *S* value. The linear trends are presented for the elastic and post-elastic stiffness’s in Figure 8a,b, respectively. It can be observed (Table 5) that the ratio *J* between *K_post_*_−*el*_ and *K_el_* varies from 4.7% to 21.7%. Therefore, the value of the stiffness in the post-elastic phase is generally from 5 to 20 times smaller than in the elastic one.

In Figure 8c, the stiffness in the post-elastic phase is related to the increase of the displacement (*T* ratio). Two types of dots were used. The red squared dots (low *S* ratio values), show low stiffness and low *T* ratio values (between 200% and 400%), i.e., the displacement *D_fail_* at failure load is 2–4 times the elastic displacement. The blue dots (high *S* ratio values) show that the stiffness in the post-elastic phase (*K_post_*_-*el*_) is higher and it decreases when the *T* ratio increases. It can be noted that the maximum value of the mid-span displacement at failure is about 11 times bigger than the corresponding value at the elastic limit.

### 4.2. Results in Terms of Energy Dissipated

From an energetic point of view, the energy content in the elastic phase is always smaller than the one in the post-elastic phase. The value of Λ*_el_*, given by the ratio between the elastic and the total energies, shows that the elastic energy varied from 4% to 26% of the total energy (Table 6).

Test results demonstrated that reinforced beams with low elastic energy values performed better in the post-elastic phases than reinforced beams with high elastic energy (Beams of Series 5a, 5b, and 5c). However, this did not occur in all beams because the post-elastic capacity also depends on the lever arm of the internal stresses and the reinforcement type. Table 6 shows the values of elastic energy (*E_el_*) and post-elastic energy (*E_p_* + *E_f_*).

**Figure 9:**
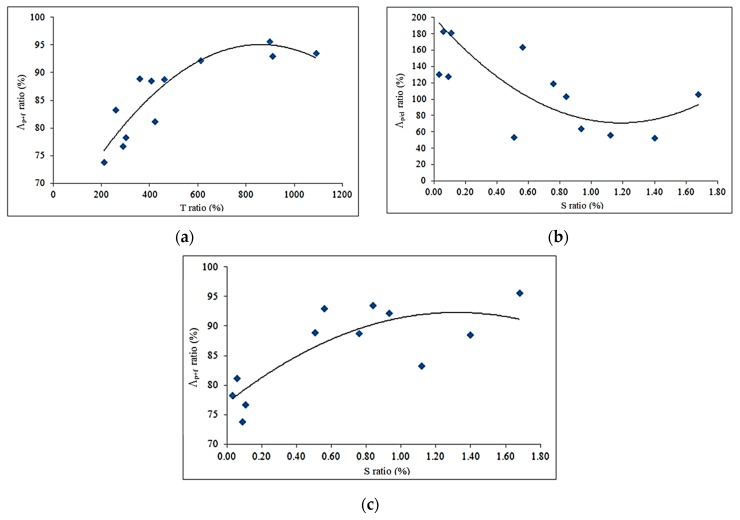
(**a**) Energy ratio Λ*_p_*_+*f*_ vs. displacement ratio; (**b**,**c**) Energy ratios Λ*_p_*_/*el*_ and Λ*_p_*_+*f*_ vs. *S* ratio.

Figure 9a shows the Λ*_p_*_+*f*_ vs. *T* ratio diagram. There is a quadratic relationship between them, showing that the more extensive the ductility is (in term of displacement) the higher the energy content is. The curve reaches a maximum when the displacement at failure is about 10 times the elastic one.

Figure 9b shows the Λ*_p_*_/*el*_ vs. area of reinforcement (S ratio) diagram. The best-fit curve to mimic the trend of the data is a quadratic equation once again. This equation has a minimum for a reinforcement area ratio of approximately 1.30%. Furthermore, by considering Figure 9c, it is possible to note that the maximum value of the post-elastic energy Λ*_p+f_* ratio corresponds to a *S* ratio of 1.30%. Further increases of the reinforcement area produce a reduction of the energy ratio Λ*_p+f_* and an increase of the ratio Λ*_p_*_/*el*_.

### 4.3. Numerical Analysis Results

**Figure 10:**
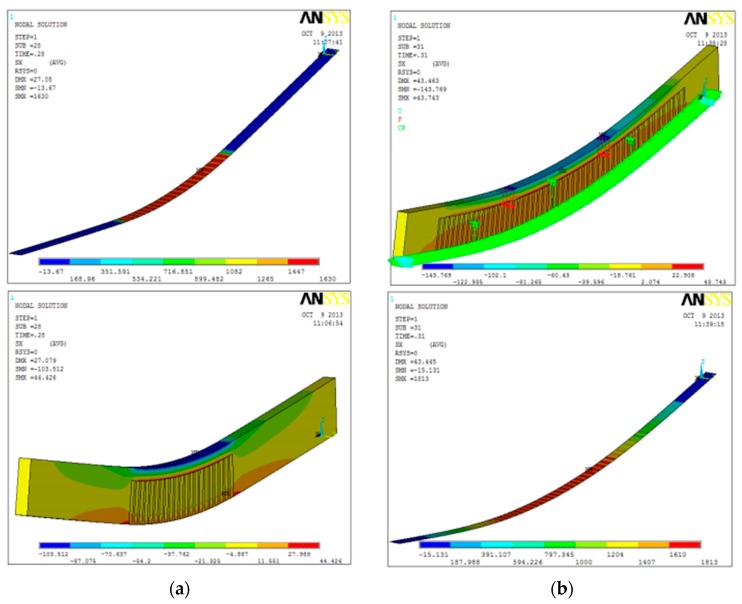
Stress patterns: (**a**) Elastic phase for the glass beam and the reinforcement; (**b**) Post-cracking phase for the glass beam and the reinforcement [Values in (MPa)].

**Figure 11:**
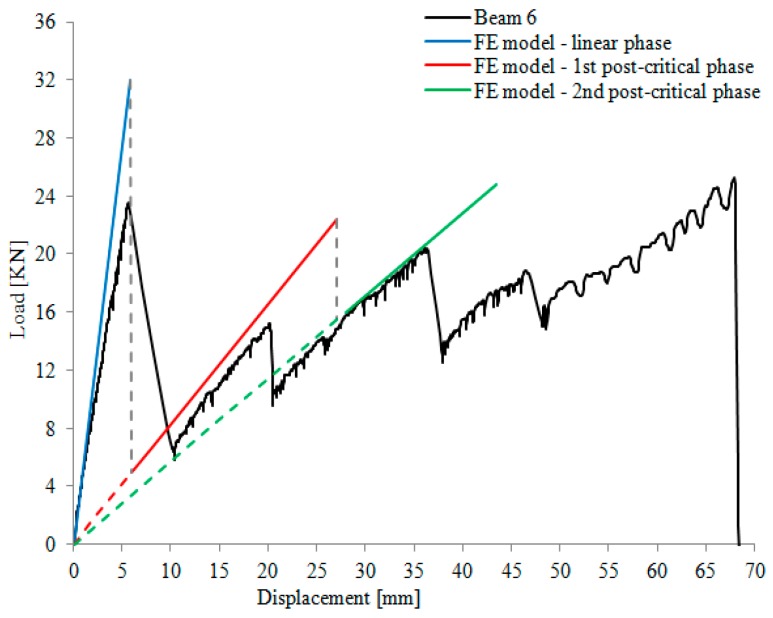
Comparison of the numerical and experimental results for a specimen from Series Beam 6.

Results of the numerical analysis are reported in Figure 10, which shows the stress patterns for both un-cracked and cracked reinforced glass beams (elastic and post-cracking phases). Figure 11 shows the response of the reinforced beam (series Beam 6). It can be noted that the numerical analysis is able to capture the experimental response of the reinforced glass beam with an acceptable error, also taking into account the non-elastic behavior of the beam, following the initial cracking. The post-elastic phase of the numerical analysis exhibits a decrease of the bending stiffness, due to the formation of the cracks. The dashed lines in Figure 11 represent the load-drops. It is not easy for the numerical model to capture the experimental behavior of the reinforced beams since the cracks progressively develop from small flaws randomly distributed in the glass material. However, such a numerical model can be used for design purposes, following an appropriate calibration.

## 5. Conclusions

The results of an experimental investigation on steel fiber-reinforced glass beams were presented in this paper. Various types of glass beams were tested with different beam dimensions, types, and quantities of steel fiber reinforcement.

Test results showed that the application of the steel fiber is not able to produce an increment of the load-capacity at the elastic limit. However, the steel fiber reinforcement can guarantee the development of a post-elastic phase and this has positive effects on the overall structural safety. Reinforced beams were able to support further bending loads after the opening of the first cracks in the glass; due to redistribution of the stresses (tensile stresses migrated from the glass to the steel fibers), the beams were able to find new equilibrium configurations. Bending capacity dropped after first cracks opened, but a subsequent load recovery was observed. In most cases, the failure load was higher than the load recorded at the end of the elastic phase. Vertical deflections highly increased during the post-elastic phase, as a consequence of glass cracking.

The post-elastic phase was studied in terms of energy dissipation. Appropriate parameters were introduced and used to consider the equivalent strain energy in the different phases of a bending test for a reinforced beam. These dimensionless parameters, taking into account the amount of dissipated energy, were used and correlated to the reinforcement area. It was concluded that the energy content in the elastic phase was always lower than the one of the post-elastic phase. The elastic energy was in the range of 4%–26% of the total energy.

Furthermore, by applying appropriate relationships, it was also possible to define a point of singularity, useful for design of steel reinforced glass beams. Test results demonstrated that an adequate reinforcement area is about 1.30% of the cross sectional area of the glass beam. This amount of reinforcement was able to maximize the energy dissipated during the post-elastic phase and to minimize the ratio between the post-elastic and elastic energy.

## Figures and Tables

**Table 1:** Physical and mechanical properties of the used epoxy resin (from producer data sheet).

Type	Epoxy, bi-Component
Density (g/cm^3^)	1.08
Consistence	Liquid
Colour	Transparent
Pot-Life a 20 °C (mass of 500 g) (min)	20
Time of complete gardening at 20 °C (days)	7
Compressive strength (MPa)	50
Flexural strength (MPa)	30
Young’s modulus (MPa)	1760

**Table 2:** Mechanical properties of the steel fibers used in the experiment.

Mechanical Properties	UHTSS Fibers	Stainless Fibers
Tensile strength (MPa)	2950	1470
Arrangement	Unidirectional	Unidirectional
Cord Diameter (mm)	1	1
Cord Density (cord/cm)	-	10
Cord Failure Load (kN)	0.8	0.7
Weight Density (kg/m^2^)	1.97	2.2
Young’s modulus (GPa)	206	73.5
Tensile elongation at failure (%)	2.3	2.0

**Table 3:** Beam dimensions and reinforcement cross sectional areas.

Beam Series	b (mm)	h (mm)	l (mm)	L (mm)	No. of Specimens	Steel Fiber Type	Reinforcement Cross Sectional Area A_steel_ (mm^2^)
Beam 1-a	16	100	1100	1000	5	Un-reinforced	-
Beam 1-b	16	100	1100	1000	5	Stainless steel	0.475
Beam 1-c	16	100	1100	1000	5	UHTSS steel	0.475
Beam 2-a	8	100	1100	1000	3	Un-reinforced	-
Beam 2-b	8	100	1100	1000	3	UHTSS steel	0.285
Beam 3	24	100	1100	1000	3	UHTSS steel	0.475
Beam 4-a	48	120	1100	1000	3	UHTSS steel	18.24
Beam 4-b	48	120	1100	1000	2	UHTSS steel	27.36
Beam 5-a	40	100	1000	900	1	UHTSS steel	7.6
Beam 5-b	40	100	1000	900	1	UHTSS steel	15.2
Beam 5-c	40	100	1000	900	1	UHTSS steel	22.8
Beam 5-d	40	100	1000	900	1	Stainless steel	19.2
Beam 5-e	40	100	1000	900	1	Stainless steel	28.8
Beam 6	48	200	3000	2800	1	UHTSS steel	27.36

b = beam’s width, h = height, l = length, L = beam span.

**Table 4:** Test results (mean values).

Beam Series	Elastic Limit Load *F_el_* (kN)	Limit Elastic Displacem. *D_el_* (mm)	Post-Elastic Load *F_post-el_* (kN)	Post-elastic Displacement *D_post-el_* (mm)	Failure Load *F_fail_* (kN)	Displacement at Failure Load *D_fail_* (mm)	*K_el_* × 10^6^ (N/m)	*K_post-el_* × 10^6^ (N/m)
Beam 1-a	6.51 *	2.57	-	-	6.51	2.57	2.54	-
Beam 1-b	7.25	3.29	2.93	6.34	5.03	13.2	2.27	0.38
Beam 1-c	5.55	2.78	2.90	5.11	3.89	8.63	2.11	0.31
Beam 2-a	2.46 *	2.30	-	-	2.46	2.30	1.08	-
Beam 2-b	2.56	1.93	1.20	4.46	1.41	7.50	1.33	0.10
Beam 3	9.54	2.96	3.20	7.04	5.11	15.5	3.24	0.23
Beam 4-a	24.5	2.32	16.6	3.20	31.4	16.5	10.8	1.12
Beam 4-b	30.6	3.33	19.5	4.40	39.2	16.9	9.18	1.58
Beam 5-a	17.5	1.54	9.31	3.19	16.0	15.5	11.4	0.54
Beam 5-b	18.8	3.03	14.2	4.00	22.6	10.8	6.21	1.23
Beam 5-c	13.8	1.30	8.84	2.14	26.2	13.0	10.6	1.61
Beam 5-d	17.5	2.74	13.7	3.57	26.3	12.6	6.41	1.39
Beam 5-e	19.9	1.97	12.2	3.43	22.5	11.0	10.1	1.36
Beam 6	23.5	5.71	5.80	10.5	25.3	67.9	4.12	0.34

* This was the failure load for un-reinforced beams.

**Table 5:** Test results (mean values).

Beam Series	*S* (%)	*R* (%)	*T* (%)	*V* (%)	*J* (%)	*Q* (%)
Beam 1-a	-	-	-	-	-	-
Beam 1-b	0.03	−30.6	301.2	40.4	16.7	171.7
Beam 1-c	0.09	−29.9	210.4	52.3	14.7	134.1
Beam 2-a	-	-	-	-	-	-
Beam 2-b	0.10	−44.9	288.6	46.9	7.5	117.5
Beam 3	0.06	−46.4	423.6	33.5	7.1	159.7
Beam 4-a	0.93	28.2	611.1	67.8	10.4	189.2
Beam 4-b	1.40	28.1	407.5	63.7	17.2	201.0
Beam 5-a	0.56	−8.6	906.6	53.2	4.76	171.9
Beam 5-b	1.12	20.2	256.4	75.5	19.8	159.2
Beam 5-c	1.68	89.9	900.0	64.1	15.2	296.4
Beam 5-d	0.50	50.3	359.9	78.3	21.7	192.0
Beam 5-e	0.76	13.1	458.4	61.3	13.5	184.2
Beam 6	0.84	7.7	1089	24.7	8.24	436.4

**Table 6:** Test results (mean values): energy parameters.

Beam Series	*E_el_* (J)	*E_p_* (J)	*E_f_* (J)	*E_p_* + *E_f_* (J)	*E_t_* (J)	Λ*_el_* (%)
Beam 1-a	8.36	-	-	-	8.36	100
Beam 1-b	11.9	15.5	27.3	42.8	54.8	21.8
Beam 1-c	7.71	9.85	12.0	21.8	29.5	26.1
Beam 2-a	2.83	-	-	-	2.83	100
Beam 2-b	2.47	4.49	3.64	8.13	10.6	23.3
Beam 3	14.1	26.0	35.0	60.9	75.1	18.8
Beam 4-a	28.4	18.2	319.1	337.3	365.7	7.77
Beam 4-b	50.9	27.0	366.7	393.4	444.3	11.4
Beam 5-a	13.5	22.1	156.2	178.3	191.8	7.04
Beam 5-b	28.5	16.0	125.7	141.7	170.3	16.8
Beam 5-c	8.96	9.55	189.9	199.5	208.4	4.30
Beam 5-d	24.0	13.0	180.1	193.0	217.1	11.1
Beam 5-e	19.6	23.4	132.0	155.4	175.0	11.2
Beam 6	67.2	69.7	893.0	962.7	1029.9	6.5
